# Seamless replacement of *Autographa californica* multiple nucleopolyhedrovirus *gp64* with each of five novel type II alphabaculovirus fusion sequences generates pseudotyped virus that fails to transduce mammalian cells

**DOI:** 10.1099/vir.0.041921-0

**Published:** 2012-07

**Authors:** Marcel Westenberg, Helen M. Soedling, Nisha Hirani, Linda J. Nicholson, Derek A. Mann, Colin T. Dolphin

**Affiliations:** 1 Pharmaceutical Science Research Division, King’s College London, Franklin-Wilkins Building, 150 Stamford Street, London SE1 9NH, UK; 2 Division of Cancer Studies, King’s College London, Guy’s Campus, London SE1 1UL, UK; 3 Liver Research Group, Institute of Cellular Medicine, 4th Floor, Catherine Cookson Building Medical School, Newcastle University, Newcastle NE2 4HH, UK

## Abstract

*Autographa californica* multiple nucleopolyhedrovirus (AcMNPV), a member of the type I alphabaculoviruses, is able to transduce and deliver a functional gene to a range of non-host cells, including many mammalian lines and primary cells, a property mediated by the envelope fusion protein GP64. AcMNPV is non-cytopathic and inherently replication deficient in non-host cells. As such, AcMNPV represents a possible new class of gene therapy vector with potential future clinical utility. Whilst not a problem for *in vitro* gene delivery, the broad tropism displayed for non-host cells is less desirable in a gene therapy vector. The fusion protein F of type II alphabaculoviruses can substitute functionally for GP64, and such pseudotyped viruses display a severely impaired capacity for non-host-cell transduction. Thus, surface decoration of such an F-pseudotyped AcMNPV with cell-binding ligands may restore transduction competence and generate vectors with desirable cell-targeting characteristics. By seamlessly swapping the native *gp64* coding sequence with each of five sequences encoding different F proteins, a set of F-pseudotyped AcMNPV was generated. This report details their relative abilities both to functionally replace GP64 in viral growth and to transduce human Saos-2 and HeLa cells. All five supported viable infections in insect cell cultures and one, the *Mamestra configurata* NPV (MacoNPV) F pseudotype, could be amplified to titres close to those of native AcMNPV. In contrast, none was able to transduce the Saos-2 and HeLa cell lines. The robust support provided by MacoNPV F in virus production makes the corresponding pseudotype a viable scaffold to display surface ligands to direct selective mammalian cell targeting.

## Introduction

The family *Baculoviridae* comprises large, rod-shaped, enveloped dsDNA viruses infective to insects belonging to the orders Lepidoptera, Hymenoptera and Diptera. Based on phylogenetic analysis of genome sequences, members of the family *Baculoviridae* are organized into four major genera: *Alpha*-, *Beta*-, *Gamma*- and *Deltabaculovirus* (Jehle *et al.*, 2006), with the former being subdivided into type I and type II alphabaculoviruses based, in part, on their use of the different major envelope fusion proteins, GP64 and fusion (F), respectively (Pearson & Rohrmann, 2002). From a historical perspective, baculovirus species have been used both as effective biological pesticides (reviewed by Szewczyk *et al.*, 2006) and, in the form of the prototype *Autographa californica* multiple nucleopolyhedrovirus (AcMNPV), as a popular gene-delivery vector for heterologous protein expression in cultured insect cells (reviewed by van Oers, 2011). Proteins expressed in such an expression system can be generated at high yield, correctly folded and with many of the post-translational modifications, such as glycosylation, associated with mammalian cells (van Oers, 2011). Some time after these applications were established, it was discovered that AcMNPV was also able to transduce and deliver a functional transgene to a range of mammalian cells both *in vitro* and *in vivo* (reviewed by Hüser & Hofmann, 2003). Demonstrated originally in primary hepatocytes and liver-derived cell lines (Hofmann *et al.*, 1995; Boyce & Bucher, 1996), the range of cells transduced by AcMNPV has since grown (see Chen *et al.*, 2011) and now includes several additional primary cell types, including hepatic stellate cells (Gao *et al.*, 2002), neural cells (Sarkis *et al.*, 2000) and pancreatic islet cells (Ma *et al.*, 2000). This unexpected competence for non-host-cell transduction has prompted researchers to design AcMNPV-based vectors for a variety of novel applications including assay development in drug discovery (Kost *et al.*, 2010), RNA interference (Nicholson *et al.*, 2005; Ong *et al.*, 2005), cancer (Wang & Balasundaram, 2010), gene therapy (Ghosh *et al.*, 2002) and engineering of stem cells in tissue regeneration (Lin *et al.*, 2010). A possible regulatory route towards the eventual clinical application of AcMNPV-based vectors has been discussed recently (Lesch *et al.*, 2011).

The broad range of non-host cells transduced by AcMNPV, together with the ability of the fusion protein GP64 to functionally replace fusion proteins from lentivirus (Schauber *et al.*, 2004) and human respiratory syncytial virus (Oomens & Wertz, 2004), indicate that GP64 acts as a promiscuous fusion protein. Whilst such lack of selectivity is not relevant for AcMNPV-mediated gene delivery to cultured mammalian cells, it is less desirable if developing AcMNPV as a potential gene therapy vector because of unwanted bystander effects. Thus, it would be advantageous if AcMNPV-based vectors could be engineered with selective cell tropisms whilst retaining facile amplification to high titre in insect cell culture and the capacity to deliver a functional cargo to non-host cells. AcMNPV GP64 is necessary for viral attachment to (Hefferon *et al.*, 1999), membrane fusion during entry into (Blissard & Wenz, 1992; Kingsley *et al.*, 1999) and subsequent budding from (Monsma *et al.*, 1996; Oomens & Blissard, 1999) host insect cells. In addition, GP64 plays a critical role in mammalian cell transduction (Tani *et al.*, 2001). A number of studies (Table 1) have demonstrated that the majority of type II alphabaculoviral F proteins, although apparently binding to a different insect cell receptor (Westenberg *et al.*, 2007), can substitute functionally for GP64 by supporting AcMNPV production in culture, albeit with reduced titres. In contrast, F-pseudotyped Δ*gp64* AcMNPV vectors are severely compromised for mammalian cell transduction (Liang *et al.*, 2005; Westenberg *et al.*, 2007, 2010; Yu *et al.*, 2009), confirming the key role that GP64 plays in non-host-cell transduction. However, the ability of F proteins to support Δ*gp64* AcMNPV in cultured host-cell entry and budding, plus the observation that they promote membrane fusion in an *in vitro* assay (Lung *et al.*, 2002), indicate that the failure of F-pseudotyped viruses to transduce mammalian cells may result from a block at virus attachment or internalization rather than endosomal escape. Thus, as others have discussed (Yu *et al.*, 2009), decorating the surface of F-pseudotyped Δ*gp64* AcMNPV vectors with, for example, cell-binding ligands or peptides represents a possible path towards generating AcMNPV-based gene therapy vectors with transductional targeting characteristics.

**Table 1.** F-pseudotyped Δ*gp64*-AcMNPV

F source*	Locus**†**	Promoter**‡**	Δ*gp64* rescue**§**	Reference(s)
SeMNPV	*polh*	*gp64*	Yes	Lung *et al.* (2002); Yu *et al.* (2009)
SeMNPV	*gp64*	*gp64*	Yes	Westenberg *et al.* (2010)
AdhoNPV	*gp64*	*gp64*	Yes	Westenberg *et al.* (2010)
LdMNPV	*polh*	*gp64*	Yes	Lung *et al.* (2002)
HearNPV	*polh*	*gp64*	Yes	Long *et al.* (2006a)
AgseGV	*polh*	*gp64*	Yes	Yin *et al.* (2008)
PlxyGV	*polh*	*gp64*	No	Lung *et al.* (2002)

* Source of F protein CDS. Type II alphabaculoviruses: SeMNPV, *Spodoptera exigua* MNPV; AdhoNPV, *Adoxophyes honmai* NPV; LdMNPV, *Lymantria dispar* MNPV; HearNPV, *Helicoverpa armigera* NPV. Betabaculoviruses: AgseGV, *Agrotis segetum* granulovirus; PlxyGV, *Plutella xylostella* GV.

† AcMNPV locus containing *F* CDS.

‡ Promoter used to drive *F* CDS.

§ Functionally substitutes for GP64.

We recently developed (Westenberg *et al.*, 2010) a modified counter-selection recombineering approach that enables us to undertake robust, seamless and unrestricted modification of the DNA sequence of the AcMNPV replicon bMON14272 (Luckow *et al.*, 1993). This modified approach was necessary to overcome, during the counter-selection step, unwanted intra-molecular rearrangements that can occur between pairs of homologous regions present in the bacmid sequence (Westenberg *et al.*, 2010). We are employing the technique as part of a strategy to generate AcMNPV-based vectors designed to deliver therapeutic genes and/or dsRNAs selectively to hepatic stellate cells in the context of liver fibrotic disease. Our initial aim is to identify, by replacing *gp64* with a range of sequences corresponding to the F-encoding ORFs from different type II alphabaculoviruses, an F-pseudotyped Δ*gp64* vector that can be cultured to high titre and lacks the promiscuous mammalian cell transduction characteristics of native AcMNPV. The envelope surface of this candidate F-pseudotyped vector will then be decorated with cell-binding ligands designed to provide selective cell targeting. Using this method, we previously reported (Westenberg *et al.*, 2010) the seamless replacement of *gp64* with *F* coding sequences (CDSs) from SeMNPV and AdhoNPV generating F-pseudotyped Δ*gp64* AcMNPV viruses that had lost the capacity to deliver a GFP transgene to mammalian cells. In the current study, we describe the construction of new Δ*gp64* AcMNPV vectors pseudotyped with five additional type II alphabaculovirus F proteins and report on their respective abilities in supporting virus production and the delivery of a reporter to mammalian cells.

## Results and Discussion

**Fig. 1.**
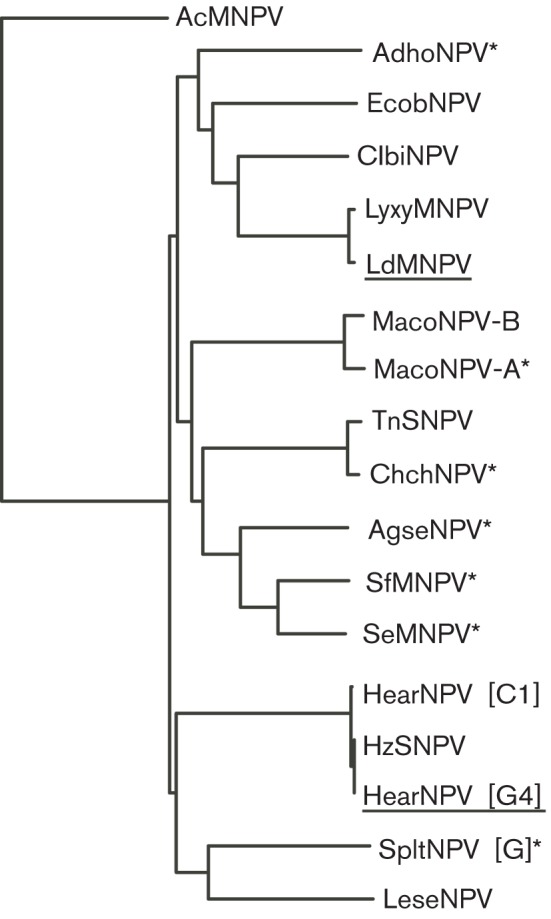
Phylogenetic analysis of type II alphabaculovirus envelope fusion proteins. A multiple sequence alignment, generated with clustal w (MacVector) (Thompson *et al.*, 1994) and optimized ‘by eye’, of representative type II alphabaculovirus F protein amino acid sequences was used to generate a phylogram by neighbour joining with systematic tie-breaking (MacVector) and rooted with type I alphabaculovirus** AcMNPV GP64. The capacity of LdMNPV and HearNPV (G4 strain) F proteins (underlined) to functionally replace AcMNPV GP64 has been examined previously by others (Lung *et al.*, 2002; Long *et al.*, 2006a). F proteins investigated by us in this, or previous (Westenberg *et al.*, 2010), studies are indicated with an asterisk. EcobNPV, *Ecotropis oblique* NPV; LyxyMNPV, *Lymantria xylina* MNPV; MacoNPV, *Mamestra configurata* NPV; TnSNPV, *Trichoplusia ni* single NPV; ChchNPV, *Chrysodeixis chalcites* NPV; AgseNPV, *Agrotis segetum* NPV; SfMNPV, *Spodoptera frugiperda* MNPV; SeMNPV, *Spodoptera exigua* MNPV; SpltNPV, *Spodoptera litura* NPV; LeseNPV, *Leucania separata* NPV.

**Fig. 2.**
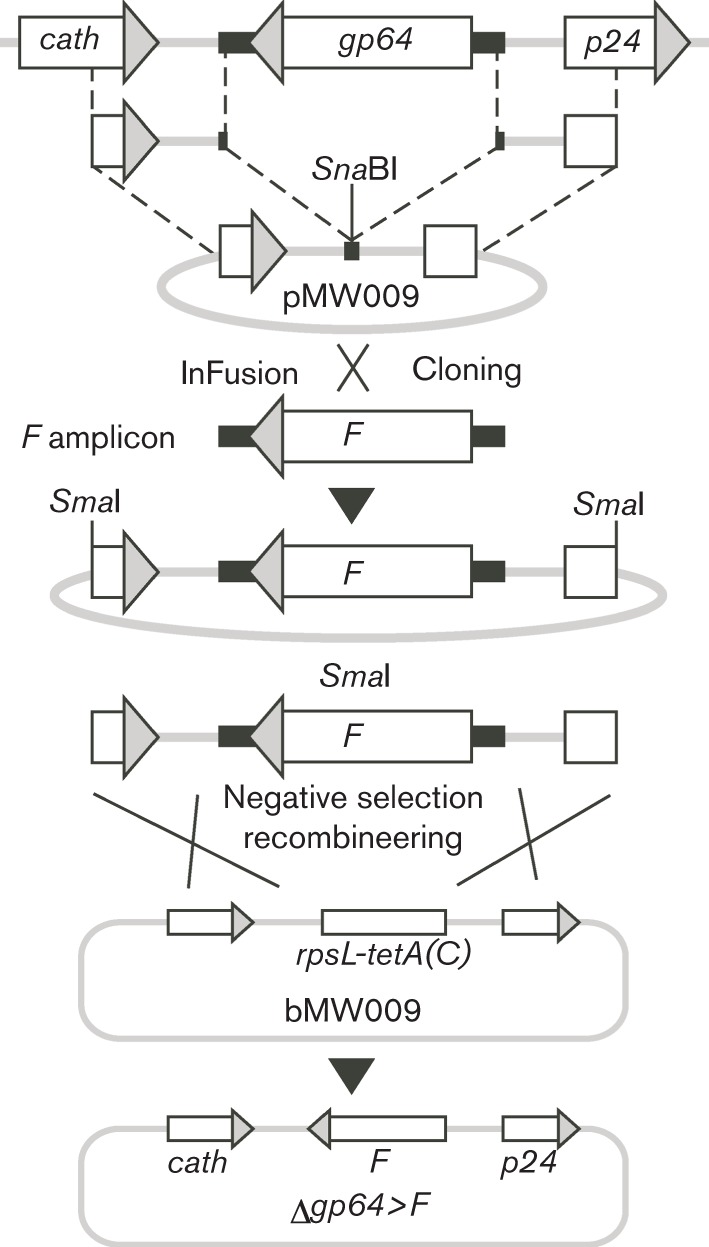
Engineering of type II alphabaculovirus F protein-pseudotyped Δ*gp64* AcMNPV bacmids. pMW009 contains ~600 bp of AcMNPV *gp64* 5′ and 3′ flanking sequences fused together with a unique, centrally located *Sna*BI restriction site. PCR amplicons, containing individual *F* CDSs flanking by short (~50 bp) AcMNPV *gp64* 5′ and 3′ flanking sequences, were introduced into *Sna*BI-linearized pMW009, via an *in vitro* recombination method (In-Fusion; Clontech), generating plasmids containing a different and discrete *F* CDS flanked by exact *gp64* 5′ and 3′ flanking sequences. These cassettes, linearized with *Sma*I, replaced the counter-selection *rpsl-tetA(C)* cassette in the Δ*gp64* AcMNPV bacmid bMW009, via λ Red-mediated recombineering with negative selection, to generate a series of bacmids in which *gp64* is seamlessly replaced by different *F* CDSs (Westenberg *et al.*, 2010).

As a guide to *F* CDS selection, we undertook a molecular phylogenetic analysis of all type II alphabaculoviral F protein sequences available at the time and, by visual examination of the resulting phylogram (Fig. 1), selected the following five previously uncharacterized species that covered the major evolutionary nodes: ChchNPV (van Oers *et al.*, 2005), SfMNPV (Harrison *et al.*, 2008), SpltNPV strain G2 (Pang *et al.*, 2001), MacoNPV-A (Li *et al.*, 2002) and AgseNPV (Jakubowska *et al.*, 2006). Using our modified recombineering protocol (Westenberg *et al.*, 2010), the *F* CDSs were PCR amplified and, following addition of extended terminal sequences homologous to the *gp64* 5′ and 3′ flanking regions, via an intermediate subcloning step, each sequence was introduced into a Δ*gp64-*bMON14272 bacmid (Fig. 2). This approach to generate F-pseudotyped AcMNPV resulted in the *F* CDS being placed in the equivalent genomic context as *gp64* and differs from the more traditional method of utilizing Tn7-mediated transposition to insert sequences into the polyhedrin (*polh*) locus (Luckow *et al.*, 1993). PCR and restriction enzyme analyses of all final bacmid sequences (Fig. S1, available in JGV Online) confirmed that our modified protocol, which employs terminal homology arms significantly longer (~600 bp) than the usually sufficient 50 bp, was both efficient – for each F pseudotype, at least three of five clones examined contained the desired *F* sequence (data not shown) – and, importantly, generated products free of the intra-molecular deletions that can otherwise commonly occur in the repetitive bMON14272 target during counter-selection recombineering (Fig. S1) (Westenberg *et al.*, 2010). The five resulting Δ*gp64*>*F* bacmids and the control Δ*gp64* bacmid were each subsequently fitted, by standard Tn7 transposition, with a GFP reporter sequence driven by a hybrid cytomegalovirus (CMV)–p10 promoter (Westenberg *et al.*, 2010), enabling facile visual monitoring of both Sf9 insect cell infection and mammalian cell transduction.

**Fig. 3.**
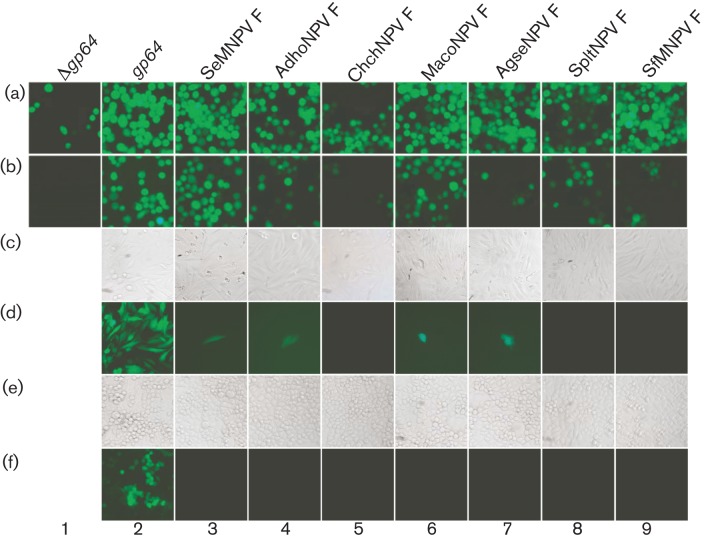
Sf9 transfection/infection and mammalian cell transduction with F-pseudotyped Δ*gp64* AcMNPV viruses. Sf9 cells (a, b) were transfected (a) with Δ*gp64* AcMPNV bacmid DNAs in which the *gp64* locus was empty (bMW024, column 1) or had been seamlessly replaced with either a rescuing native *gp64* CDS (bMW033, Δ*gp64*>gp64 AcMNPV, column 2), or the major envelope fusion CDS *F* from SeMNPV (bMW011, column 3), AdhoNPV (bMW036, column 4), ChchNPV (bMW023, column 5), MacoNPV (bMW021, column 6), AgseNPV, bMW035, column 7), SpltNPV (bMW034, column 8) or SfMNPV (bMW022, column 9) and, at 5 days post-transfection, inspected by fluorescence microscopy for GFP expression. An aliquot of the medium from each culture was used to infect a new batch of Sf9 cells (b) and, 3 days p.i., inspected for GFP expression. Cultures of either Saos-2 (c, d) or HeLa (e, f) cells were cultured (1 h, 28 °C) with clarified, filter-sterilized medium (500 µl) containing either the GP64-rescue (column 2) or F-pseudotyped (columns 3–9) virus and cells were visualized using bright-field (c, e) or fluorescence microscopy (d, f) at 2 days after incubation. For both cell types cultured with an F-pseudotyped virus, if fluorescence microscopy revealed a rare GFP-expressing cell(s), this field of view is provided. A black image indicates no GFP-expressing cells were detected. Magnification ×20. For bacmid construction, see this study, except for bMW033, bMW011 and bMW036 (Westenberg *et al.*, 2010).

A transfection/infection assay (Fig. 3a, b) confirmed the ability of all the F pseudotypes to mediate viable infections from transfected Sf9 cells, confirming that all the F proteins investigated in this present study could functionally replace GP64. Thus, to date, it appears that the ability to functionally replace GP64 may be a universal feature common to all F proteins from type II alphabaculoviruses (Table 1). In contrast, of the two F proteins investigated from betabaculoviruses, only the fusion protein from AgseGV was demonstrated to be functionally analogous to GP64 (Yin *et al.*, 2008) (Table 1).

We used a semi-quantitative assay as a means of directly comparing the ability of each of the five F-pseudotyped viruses generated in this study, plus two generated previously and the Δ*gp64*>*gp64* rescue vector (Westenberg *et al.*, 2010), to functionally replace GP64. All viruses were amplified in Sf9 cells under equivalent conditions of culture and m.o.i., and titres were determined at 5 days post-infection (p.i.). All F-pseudotyped bacmids and the *gp64* rescue bacmid were able to support virus production in culture (Table S1) with titres ranging from 1.9×10^6^ to 1.5×10^8^ p.f.u. ml^−1^ for the ChchNPV (vMW023) and MacoNPV (vMW021) F pseudotypes, respectively. In comparison, the AcMNPV GP64 rescue virus (vMW033) was amplified to ~10^9^ p.f.u. ml^−1^. Thus, although the titre was tenfold less than the GP64 rescue virus, the MacoNPV F-pseudotyped virus amplified to a useable concentration and this pseudotype has become our working vector for further development. Subsequently, in order to generate sufficiently concentrated virus stocks to enable mammalian cell transduction at virus : cell ratios close to 100, additional amplifications, with empirical combinations of starting cell densities and m.o.i. (data not shown), were carried out for those pseudotypes, such as ChchNPV F, that produced low titres in the first, controlled amplification. These provided working virus stocks for all F pseudotypes of ≥2.0×10^7^ p.f.u. ml^−1^ apart from ChchNPV and SpltNPV, which remained difficult to amplify to titres above those first recorded (Table S1).

Having generated working viral stocks, we then investigated whether any of the F pseudotypes were able to transduce either of the human cell lines Saos-2 and HeLa, which are readily transduced by native AcMNPV. Following incubation of Saos-2 cells with SeMNPV, AdhoNPV, MacoNPV or AgseNPV F-pseudotyped virus, fluorescence microscopy identified only very occasional, discrete GFP-expressing cells in each case (Fig. 3d). Apart from these isolated cells, no other GFP-expressing Saos-2 cells were observed. In HeLa cells, the situation was even more marked, as extensive searching failed to observe even occasional GFP-expressing cells after incubation with any of the F-pseudotyped viruses (Fig. 3f). As expected, the GP64 rescue vector transduced both Saos-2 and HeLa cells with efficiencies that were routinely >90 % (Fig. 3 and data not shown). Therefore, although all the F proteins tested here were able to substitute functionally for GP64 and support virus generation, the loss of the native envelope fusion protein essentially abolished the capacity for non-host-cell transduction, confirming the key role that GP64 plays in this process.

**Fig. 4.**
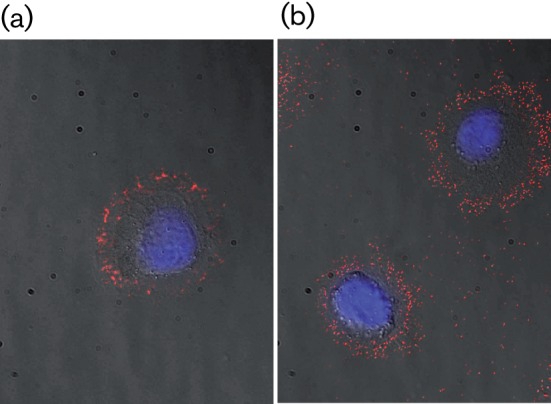
Representative images of MacoNPV F-pseudotyped AcMNPV and a *gp64* rescue vector binding to Saos-2 cells. Saos-2 cells, incubated with either vMW033 (Δ*gp64*>*gp64* rescue vector) (a) or vMW021 (Δ*gp64*>MacoNPV *F*) (b), were fixed, blocked and immunolabelled for AcMNPV VP39 with anti-VP39 mAb and Cy3-conjugated goat-anti-mouse antibody immediately after incubation. Nuclei were stained with Hoechst 33342 (blue).

A number of studies have reported on investigations into the attachment and entry mechanisms involved during mammalian cell transduction with native GP64-equipped AcMNPV. Whilst early studies indicated roles for simple electrostatic interactions (Duisit *et al.*, 1999) and phospholipids (Tani *et al.*, 2001), later reports proposed the involvement of clathrin-mediated endocytosis (Long *et al.*, 2006b) and macropinocytosis (Matilainen *et al.*, 2005). More recently (Laakkonen *et al.*, 2009), it has been suggested that neither of these latter mechanisms is significantly involved and that, instead, entry of AcMNPV into mammalian cells is mediated via a phagocytosis-like uptake process involving large plasma membranous invaginations associated with raft structures. Interestingly, experimental evidence has also been provided (Dong *et al.*, 2010), demonstrating that incubation at low pH promotes AcMNPV uptake into both host Sf9 and non-host mammalian cells via an endosome-independent pathway involving direct membrane fusion at the cell surface. In contrast to these studies involving AcMNPV, there is little, if any, data available that sheds light on the block in mammalian cell transduction observed with F-pseudotyped AcMNPV that we, and others, have reported. As a first step towards this, we undertook a preliminary capsid immunolabelling experiment to investigate the fate of MacoNPV F-pseudotyped AcMNPV following incubation with Saos-2 cells (Fig. 4). Soon after incubation, immunostaining at the cellular periphery was observed with both the MacoNPV F-pseudotyped virus and a Δ*gp64*>*gp64* rescue virus, indicating that the former bound to the cell membrane. The lack of subsequent GFP expression in the cells bound by this pseudotype suggested a block at some subsequent point in the route normally followed by GP64-equipped virus. Further experiments are required to expand and extend this observation including the use of additional pseudotypes and cell lines and investigations to examine the effect of lowering the pH during cell–virus incubation.

The modified counter-selection recombineering method described by us previously (Westenberg *et al.*, 2010) that enables precise and seamless modification of the AcMNPV genome has been further validated by the work detailed in the present report. The optimized methodology will enable others to undertake targeted modifications to the AcMNPV genome ranging from the types of seamless CDS swaps reported here down to subtle, single base-pair deletions, insertions or changes. We are employing this approach to furnish the surface of MacoNPV F-pseudotyped AcMNPV with cell-binding ligands designed to provide desired cell-targeting properties. If successful, such a strategy would represent an advance towards creating potentially clinically useful AcMNPV-based gene therapy vectors.

## Methods

### 

#### Phylogenetic analysis.

Type II alphabaculovirus F and AcMNPV GP64 amino acid sequences were aligned using clustal w (Thompson *et al.*, 1994), within MacVector sequence analysis software (MacVector Inc.), and the resulting multiple sequence alignment file was optimized by eye. Using this file as input, phylogenetic analysis (again within MacVector) was undertaken, via neighbour joining with systematic tie-breaking, and the resulting phylogram was rooted with GP64.

#### Microscopy.

All bright-field and fluorescence microscopy was undertaken on an Olympus BX61 motorized microscope equipped with appropriate filter sets. Post-acquisition image analysis was performed with CellSens (Olympus) and ImageJ (NIH) packages.

#### Plasmid construction and bacmid recombineering.

Sequences encoding the F proteins of ChchNPV, SfMNPV, SpltNPV, MacoNPV-A and AgseNPV were PCR amplified (Phusion; NEB) using 5 ng of the corresponding viral genomic DNA as template and the respective PAGE-purified oligonucleotide (ODN) pairs 6021a/6022a, 6017a/6018a, 6108/6109, 6104/6105 and 6013a/6014a (Table S2). Each resulting amplicon contained the individual *F* CDS flanked by 62 bp of 5′ and 46 bp of 3′ sequence equivalent to regions immediately flanking the *gp64* CDS of AcMNPV. The five amplicons were each cloned directly into *Sna*BI-linearized pMW009, the construction and utility of which has been described elsewhere (Westenberg *et al.*, 2010), via an *in vitro* recombination system (In-Fusion; Clontech) according to the manufacturer’s protocol, generating plasmids pMW025 (ChchNPV *F*), pMW026 (SfMNPV *F*), pMW031 (SpltNPV *F*), pMW030 (MacoNPV *F*) and pMW032 (AgseNPV *F*) (Table S3). Following DNA sequencing to confirm construct fidelity, the cloned sequences were released from their respective constructs by restriction with *Sma*I, generating *F* CDS-containing, recombineering-ready cassettes, which were gel purified and quantified by visualization against a DNA molecular mass ladder (2-log ladder; NEB). In addition, a control, Δ*gp64 Sma*I restriction fragment, containing only 5′ and 3′ *gp64* flanking sequences, was released from pMW009.

Each cassette, now equipped with ~600 bp flanking homology arms, was used to replace the *rpsL*-*tet(A)* counter-selection cassette (RT cassette) (Stavropoulos & Strathdee, 2001) in bacmid bMW009, in which the AcMNPV *gp64* CDS has been directly replaced by the RT cassette, via counter-selection recombineering, as described previously (Westenberg *et al.*, 2010). Briefly, aliquots (~0.1 ml) of bMW009-containing *Escherichia coli* MW001 cells (Westenberg *et al.*, 2010), induced for phage λ Red activities and made electrocompetent, as described previously (Dolphin & Hope 2006), were electroporated with one of the five *F* CDS-containing cassettes or the Δ*gp64*, non-*F* CDS-containing control cassette (*Sma*I fragment of pMW009) (500 ng each), recovered (in SOC medium without Mg^2+^ at 32 °C with shaking at 220 r.p.m. for 2.5 h) and serially diluted (in M9 salts), and aliquots (50 µl) were spread on selective [containing 50 µg kanamycin (Kan) ml^−1^, 500 µg streptomycin (Sm) ml^−1^] or non-selective (containing 50 µg Kan ml^−1^) NSLB agar (Stavropoulos & Strathdee, 2001) plates and incubated at 32 °C for 48 h. For each cassette, five discrete Sm^R^ colonies were restreaked onto fresh Kan/Sm-selective plates, incubated at 32 °C for 48 h and subjected to colony PCR to identify clones in which the RT cassette had been replaced with an *F* CDS. Subsequent restriction digestion analyses of the resulting PCR products were performed to confirm the integrity of the *F* CDS replacement sequence (Fig. S1). Single bacmid clones, containing the correctly inserted ChchNPV, SfMNPV, SpltNPV, MacoNPV or AgseNPV *F* CDS or control sequence were named, respectively, bMW019, bMW018, bMW026, bMW017, bMW027 and bMW020 (Table S3). Bacmid DNAs were isolated and subjected to restriction mapping together with bMON14272, bMW009 and the previously generated bacmids bMW010, bMW028 and bMW025 (Westenberg *et al.*, 2010) in which the native *gp64* CDS was replaced by, respectively, SeMNPV *F*, AdhoNPV *F* and rescue AcMNPV *gp64* CDSs (Fig. S1). Finally, each of the nine homologous regions (*hr1*, *hr1a*, *hr2*, *hr2a*, *hr3*, *hr4a*, *hr4b*, *hr4c* and *hr5*) was PCR amplified with flanking ODNs, as described previously (Westenberg *et al.*, 2010), and compared electrophoretically with equivalent PCR products amplified from bMON14272 (data not shown).

Following transformation with the Tn7-transposase helper plasmid pMON7124 (Luckow *et al.*, 1993), bacmids bMW017, bMW018, bMW019, bMW026, bMW027 and bMW020 in *E. coli* MW001 received, via Tn7-mediated transposition (Bac-to-Bac; Invitrogen), the CMV_PROM_p10_PROM_-eGFP dual insect/mammalian GFP reporter sequence from pMW005 (Westenberg *et al.*, 2010), generating the respective bacmids bMW021, bMW022, bMW023, bMW034, bMW035 and bMW024 (Table S3).

#### Virus generation and mammalian cell transduction.

Sf9 insect cells were passaged in shaking cultures (30 ml, 90 r.p.m., 28 °C) in serum-free medium (Sf900II SFM; Invitrogen) supplemented with 2 % (v/v) FBS. For virus generation, Sf9 cells (1.0×10^6^) grown in monolayers were transfected (Cellfectin; Invitrogen) with bacmid DNA (~1 µg), cultured (5 days, 28 °C) and inspected for GFP expression by fluorescence microscopy. An aliquot (500 µl) of the clarified (10 min, 1000 ***g***), filter-sterilized (0.45 µm) medium was used to infect a new batch of Sf9 cells (1.0×10^6^). At 3 days p.i., cells were inspected for GFP expression. Subsequently, cells were passaged every 4–5 days until all cells were infected, at which point titres were determined by an end-point dilution assay (O’Reilly *et al.*, 1992). Quantitative determination of relative titre generation was performed by infecting Sf9 cells (1.0×10^6^ cells ml^−1^) in a shaking culture (30 ml, 90 r.p.m.) at an m.o.i. of 0.05. Cultures were harvested at 5 days p.i. and centrifuged (10 min, 1000 ***g***) and the supernatants were filter sterilized. Titres were determined and the supernatants were stored at 4 °C. Individual bacmid constructs generated the corresponding ‘v’-prefixed virus (Table S3).

Saos-2 and HeLa cells were maintained (37 °C, 5 % CO_2_) in Dulbecco’s modified Eagle’s medium (DMEM), supplemented with 10 % FBS. Cells were seeded (1.0×10^5^) in 24-well plates and, after incubation (24 h), the medium was replaced with 500 µl clarified, filter-sterilized virus-containing Sf900II SFM resulting in virus : cell ratios of 50 (vMW022 and vMW023) or 100 (vMW011, VMW021, vMW033, vMW034, vMW035 and vMW036). After 1 h incubation at 28 °C with gentle shaking, the medium was aspirated, the cells were washed and fresh DMEM or Eagle’s minimal essential medium was added as appropriate. GFP expression was visualized in transduced cells by fluorescence microscopy (magnification ×20) at 48 h post-transduction.

#### Immunohistochemistry.

Soas-2 cells (1.0×10^5^), seeded on poly-l-lysine-coated coverslips placed in the wells of a 24-well plate, were cultured for 14 h, as described above, cooled to 4 °C and incubated with vMW033 (Δ*gp64*>*gp64* rescue vector) or vMW021 (MacoNPV F-pseudotyped AcMNPV) for 1.5 h at 4 °C with gentle shaking at a virus : cell ratio of 500, after which they were washed twice with ice-cold PBS and fixed immediately (4 % formaldehyde, room temperature, 20 min). After fixation, the cells were washed three times with PBS, permeabilized with 0.2 % Triton X-100 in PBS for 10 min, washed again three times with PBS and blocked with 10 % goat serum in PBS for 2 h. The cells were then incubated for 14 h at 4 °C with an anti-VP39 mAb (Whitt & Manning, 1988) diluted 1 : 200 in 1 % goat serum in PBS, washed three times with PBS and incubated for 1 h at 37 °C with Cy3-conjugated goat anti-mouse antibody (Catlag Laboratories) diluted 1 : 200 in 1 % goat serum in PBS. As controls for specific antibody binding, mock-transduced cells, incubated with both antibodies, and transduced cells, incubated with the secondary antibody only, were used. Cells were finally washed twice with PBS, incubated for 30 min with Hoechst 33342 (10 µM in PBS; Sigma) and washed again twice with PBS. Coverslips were mounted in Fluorescence Mounting Medium (Dako) and the cells imaged (magnification ×100).
